# The TSPO Ligands MGV-1 and 2-Cl-MGV-1 Differentially Inhibit the Cigarette Smoke-Induced Cytotoxicity to H1299 Lung Cancer Cells

**DOI:** 10.3390/biology10050395

**Published:** 2021-05-02

**Authors:** Nidal Zeineh, Rafael M. Nagler, Martin Gabay, Fadi Obeid, Meygal Kahana, Abraham Weizman, Moshe Gavish

**Affiliations:** 1 Department of Neuroscience, The Ruth and Bruce Rappaport Faculty of Medicine, Technion Institute of Technology, Haifa 31096, Israel; nidalz@campus.technion.ac.il (N.Z.); nagler@tx.technion.ac.il (R.M.N.); mgabay@campus.technion.ac.il (M.G.); fadiobeid@campus.technion.ac.il (F.O.); miglc@campus.technion.ac.il (M.K.); 2 Research Unit, Geha Mental Health Center and Laboratory of Biological Psychiatry, Felsenstein Medical Research Center, Petah Tikva 4910002, Israel; weizmana@gmail.com; 3 Departments of Psychiatry, Physiology and Pharmacology, Sackler Faculty of Medicine, Tel Aviv University, Tel Aviv 69978, Israel

**Keywords:** TSPO, TSPO ligands, cigarette smoke, mitochondrial membrane potential, ROS, apoptotic markers, cell death

## Abstract

**Simple Summary:**

In this study, we investigated the impact of CS on various TSPO-related mitochondrial processes, and the protective ability of our novel TSPO ligands against such CS-induced cellular damages. Our results support the previously reported role of TSPO in apoptotic cell death. Moreover, the present data demonstrate the protective effect of our TSPO ligands against CS-induced cellular damage.

**Abstract:**

TSPO is involved in cigarette smoke (CS)-induced cellular toxicity, which may result in oral and pulmonary diseases and lung cancer. H1299 lung cancer cells were exposed directly to CS. The H1299 cells were pretreated with our TSPO ligands MGV-1 and 2-Cl-MGV-1 (Ki = 825 nM for both) at a concentration of 25 µM 24 h prior to CS exposure. Cell death and apoptotic markers were measured, in addition to TSPO expression levels, ATP synthase activity, generation of reactive oxygen species (ROS), depolarization of mitochondrial membrane potential (ΔΨm), cAMP and LDH levels. Pretreatment with MGV-1 and 2-Cl-MGV-1 (25 µM), 24 h prior to CS exposure, differentially attenuated the CS-induced cellular insult as well as cell death in H1299 lung cancer cells. These protective effects included prevention of ATP synthase reversal, ROS generation, depolarization of the mitochondrial membrane and elevation in LDH. The preventive efficacy of 2-Cl-MGV-1 was superior to that achieved by MGV-1. Both ligands did not prevent the elevation in cAMP. These findings may indicate a mild protective effect of these TSPO ligands in CS-related pulmonary and keratinocyte cellular pathology.

## 1. Introduction

Cigarette smoke (CS) is a main cause of various human diseases and associated with high mortality rates [1]. CS is responsible for about 90% of pulmonary diseases and lung cancer cases [2] as well as oral diseases [3,4]. The direct insult of CS contributes to the development of the various diseases, including oral cancer, pulmonary cancer, emphysema and chronic obstructive pulmonary disease (COPD). CS also contributes to the development of cancer in distant regions as well [5,6,7,8]. The most effective treatment for CS-related diseases is smoking cessation; however, several irreversible damages persist [9].

The 18 kDa translocator protein (TSPO) is mainly located in the outer membrane of the mitochondria, which can be found in nuclear and perinuclear sites as well [10]. A previous study noted the association between CS exposure and its effect on TSPO binding affinity in both in vivo and in vitro models [11]. TSPO is involved in various cellular functions that are relevant to cigarette smoking, such as oxidative stress, programmed cell death, inflammation, and cancer [12,13,14]. Previous studies described the association between TSPO and cancer [15,16,17,18,19]. One study demonstrated a significant difference in the density of TSPO binding sites between low- and high-grade tumors as compared to normal human cortex; also, a significant difference was observed in the density of TSPO binding sites between the low-grade tumors as compared to high-grade tumors [20]. However, another binding study did not show a difference in B_max_ between high- and low-grade tumors when density of TSPO binding sites was assessed in tissue homogenates [21].

The role of TSPO in CS-related apoptotic pathway was demonstrated previously [22]. Various inflammatory molecules including tumor necrosis factor alpha (TNF-α) and different interleukins (e.g., IL-1β and IL-6) are produced in pulmonary epithelial cells when exposed to CS [23]. In addition to these inflammatory mediators, the transforming growth factor-β (TGF-β) is also produced by the epithelial cells, eventually leads to lung fibrosis and, subsequently, leads to programmed cell death to commence, which results in pulmonary diseases [23]. Apoptosis may be initiated by caspases 8 and 9, subsequently activating the executioner caspases 3 and 7 [24,25]. The Bcl-2 family proteins are essential regulators of apoptotic processes [26,27,28,29]. Bax/Bak participate in the release of cytochrome-c from the mitochondria and, thus, initiating the apoptotic cascade [22,30], while the anti-apoptotic Bcl-2 proteins act as suppressors of programmed cell death [31]. This anti-apoptotic effect subsequently leads to inhibition of the caspase activity [32]. In addition, the cyclic adenosine monophosphate (cAMP) may modulate cell death, as it may play an anti-apoptotic or a pro-apoptotic role [33], and may be targeted in the treatment of different cancers [34,35].

In case of apoptotic cell death, the nuclear DNA becomes more densely condensed, which is not the case of necrotic cell death. In contrast to apoptotic cells, the nuclei in healthy cells are spherical with uniformly dispersed DNA across the nucleus [36]. Condensed DNA can discriminate between apoptotic cells and healthy or necrotic cells.

In the current study, we aim to examine the protective capacity of our TSPO ligands exhibiting low affinity, MGV-1 and 2-Cl-MGV-1, against the TSPO-related mitochondrial damages caused by CS exposure, which lead to cell death, mainly apoptotic cell death in H1299 lung cancer cells.

## 2. Materials and Methods

### 2.1. Cells and Study Design

H1299 cells derived from a human non-small-cell lung carcinoma and characterized by a deletion of the p53 tumor suppressor gene were used in the study. The effect of CS and of TSPO ligands on CS-induced changes were tested on H1299 cells as a non-small-cell lung carcinoma cell model.

The H1299 lung cancer cells were maintained according to the American Type Culture Collection (ATCC) instructions: the culture medium consisted of RPMI with high glucose and without L-glutamine and sodium pyruvate, complemented with fetal bovine serum (10%), glutamine (2%) and gentamycin (50 mg/mL). The cells were grown at 37 °C in 5% CO_2_ for 48 h to reach 80% confluency, followed by 24 h of incubation with either the TSPO ligand MGV-1 or 2-Cl-MGV-1 in serum-deprived medium (containing 0.5% fetal bovine serum, along with the other previously mentioned components). In case of simultaneous treatment, the ligands were applied immediately at the time of placement of the plates into the CS chamber, while in case of post-CS exposure treatment the ligands were applied 1 h after CS exposure. The two ligands, MGV-1 and 2-Cl-MGV-1 (both at 25 µM), were chosen according to previous dose-response studies [37,38].

Following incubation with TSPO ligands at a concentration of 25 µM, the cells were exposed for 60 min to CS. The groups included in the various experiments were negative control group with serum-deprived medium with 1% ethanol as a vehicle exposed to fresh air; groups of cells pre-treated with the ligands and exposed to fresh air; positive control group of cells in vehicle-containing serum-deprived medium (similar to the negative control group) and exposed to CS; groups of cells pre-treated with the ligands and exposed to CS. In each experiment, the same number of cells was seeded initially for all the experimental groups, thus all procedures were applied to the same number of cells initially seeded.

### 2.2. Ligand Synthesis

The synthesis of TSPO ligands were performed according to the methods described by the state-of-art, as previously described [38]. The reactions of this process were monitored using analytical thin layer chromatography (TLC) and performed in flame-dried glassware under argon atmosphere. To measure the absorbance at a wavelength of 254 nm, silica gel-coated glass plates with F254 indicator were used. Afterwards, different spectrometers were used to record ^1^H and ^13^NMR as well as to report chemical shifts. Achieving high-resolution mass spectrum (HRMS) was done by using an atmospheric pressure photoionization source (APPI). In addition, column chromatography and production of 2-Arylquinazolin-4-ol were done. As described previously, further handling of the reaction mixture was performed, then, silica gel chromatography was used to purify the resultant product, and finally, DCM/ethylacetate/n-pentane was used to recrystallize it [38].

### 2.3. Exposure of H1299 Cells to Cigarette Smoke

**Scheme 1:**
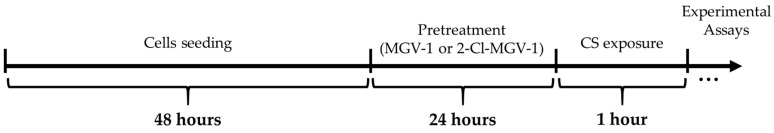
The study design. CS = cigarette smoke.

Initially, H1299 cells were seeded in culture medium and incubated for 48 h in appropriate size dishes (96-well plates with 200 μL of medium per well and 6-well plates with 3 mL of medium per well, resulting in a layer of medium covering the cells of approximately 3 mm for both), until the desired confluency was reached (80% confluency). This was followed by pretreatment application for 24 h by replacing the full medium with 1% ethanol-containing serum-deprived medium and ligands containing starvation medium. Subsequently, this was followed by 60 min of CS exposure without changing the medium (Scheme 1).

Appropriate dishes containing the cell groups submerged in medium with various treatment types were exposed to CS in a chamber sealed by vacuum generation and connected with a sidearm to which the cigarette was attached. The cigarettes used were filtered “Time” cigarettes (Dubek, Petah Tikva, Israel) containing 14 mg of tar and 0.9 mg of nicotine per cigarette. Low pressure inside the chamber was obtained using a vacuum pump. The cigarette was lit, and the smoke was sucked into the sealed chamber, as described previously [39]. After the entire cigarette was burned, the experimental plates remained exposed for 15 min to CS within the chamber, then the cigarette was replaced. In this fashion, cigarettes were lit every 15 min for a total period of 60 min. Control groups were simultaneously exposed to fresh air inside another chamber.

### 2.4. TSPO and cAMP Levels Measurement Using CyAN ADP

Steps of samples preparation were performed on ice. Following CS exposure, the cells from all plates were detached using trypsin and collected in falcons along with their medium, then centrifuged for 5 min at 660× *g*. Four per cent paraformaldehyde was used for fixation of the pellets for 10 min followed by washing using PBS without Ca^+2^ and Mg^+2^. Then, 800 μL of PBS with 0.2% tween (PBS-T) was added to the pellets and incubated for 10 min. The samples were washed again with PBS and followed by overnight incubation at 4 °C in 100 μL of PBS-T containing 3% BSA and anti-TSPO or anti-cAMP antibody (1:100 dilution according to the manufacturer’s instructions) (Abcam, Cambridge, UK). On the following day, the cells were washed with PBS and re-suspended in 100 μL of PBS-T containing 3% BSA and Alexa Fluor 488 AffiniPure Goat Anti-Rabbit IgG (Jackson Immunologicals, West Grove, PA, USA). FACS machine CyAN ADP (Beckman Coulter, Brea, CA, USA) was used to measure the mean fluorescence intensity. The data were analyzed using FlowJo (FlowJo LLC, Ashland, OR, USA). In the current FACS experiments, the negative control was cells not exposed to CS (exposed only to fresh air), while the positive control was cells exposed to CS. All experiment included unstained samples of cells in order to calibrate the fluorescence intensity prior to the MFI reading of each experimental group. This FACS method for quantification allows the single cell-based quantification of protein levels; thus, it allows the measurement of fluorescence levels per single cell.

### 2.5. ADP/ATP Ratio

For ADP/ATP ratio assay, cells were seeded in 96-well white plates, followed by application of the vehicle or the ligands and exposure to CS or fresh air. The ADP/ATP ratio assay kit was used according to the manufacturer’s protocol (MAK135; Sigma-Aldrich, St. Louis, MO, USA), as previously described [40]. Then, ADP/ATP ratio was measured using ELISA. Infinite M200 Pro plate reader was used to measure luminescence levels (Tecan, Männedorf, Switzerland). ADP/ATP ratio was calculated according to the formula given by the manufacturer.

### 2.6. Cellular ROS/Superoxide Detection Assay

Oxidative stress and superoxide levels were measured using the ROS/Superoxide detection assay kit according to the manufacturer’s instructions (Abcam, Cambridge, UK). The samples were washed using 1× wash buffer. Further treatment was performed by applying 100 μL/well of ROS/Superoxide Detection Solution, containing the treated cells. Cells were stained and incubated for 60 min at 37 °C in the dark. Bottom reading without removing the detection mix was performed, and fluorescence was measured using Infinite M200 Pro plate reader (Tecan, Männedorf, Switzerland) with standard fluorescein (excitation at a wavelength of 488 nm and emission at a wavelength of 520 nm) filter set.

### 2.7. Depolarization of the Mitochondrial Membrane Potential (ΔΨm)

JC-1 (5,5′,6,6′-tetrachloro-1,1′,3,3′-tetraethylbenzimidazolylcarbocyanine-chloride) was used to assay depolarization of the Δψm, as described previously [8]. The Δψ_M_ is an important parameter of mitochondrial function and, thus, can be used as an indicator of cell health. JC-1 is a lipophilic, cationic dye that can selectively enter into the healthy mitochondria and reversibly changes color from green (emission at 527 nm) to red (emission at 590 nm) as the membrane potential increases. A decrease in the red/green fluorescence intensity ratio is an indication for Δψ_M_ depolarization [8].

### 2.8. Cell Death Assays

#### 2.8.1. Lactate Dehydrogenase (LDH) Activity

Cytotoxicity was measured by assessment of LDH levels released to the cells containing media. This enzyme is released from the cells when the cell membrane is compromised, such as in necrotic cell death as well as in the late stages of apoptotic cell death [41]. According to the manufacturer’s protocol, Cytotoxicity Detection Kit (LDH) (Roche pharmaceuticals, Basel, Switzerland) with an absorbance at 492 nm wavelength and a reference wavelength of 620 nm was applied, with the use of the Spectrophotometer Zenyth 200 (Anthos, Eugendorf, Austria), and the results were normalized according to the formula given by the manufacturer.

#### 2.8.2. Apoptosis Levels

Apoptotic cell death levels were measured using apoptosis/necrosis detection kit according to the manufacturer’s protocol (Abcam, Cambridge, UK). Cells were grown in 12-well plates for 48 h, followed by pretreatment with our TSPO ligands MGV-1 and 2-cl-MGV1 and, on the next day, exposed for 60 min of CS. Following CS exposure, the cells were trypsinized, centrifuged and collected in an assay buffer. Then, the Apopxin green indicator was added to the cells and incubated for 45 min. A FACS instrument, CyAN ADP (Beckman Coulter, Brea, CA, USA), was used to measure the mean fluorescence intensity. The data were analyzed using FlowJo software (FlowJo LLC, Ashland, OR, USA).

### 2.9. Caspase-3, Caspase-8 and Caspase-9 Levels

Cells were seeded and pretreated with or without the TSPO ligands in 96-well plates, followed by exposure to fresh air or CS. The apoptotic markers caspase-3, caspase-8 and caspase-9 were measured using the Multiplex Activity Assay Kit (Abcam, Cambridge, UK), according to the manufacturer’s protocol. Fluorescence was measured using Infinite M200 Pro plate reader (Tecan, Männedorf, Switzerland) with specific wavelengths: Caspase 3 at Ex/Em = 535/620 (red), Caspase 8 at Ex/Em = 490/525 (green), Caspase 9 at Ex/Em = 370/450 nm (blue).

### 2.10. Microscopic Imaging

#### 2.10.1. TSPO Levels

Following 60 min of CS exposure of the pretreated experimental groups, the cells were fixed with 4% paraformaldehyde for 10–15 min, washed with 500–600 µL of PBS without Ca^+2^ and Mg^+2^, then followed by incubation for 10 min on ice in 500 μL of PBS-T (0.2% tween) and then washed again with PBS. The samples were incubated overnight in 500 μL of PBS-T with 3% BSA and containing anti-TSPO antibody (1:1000) (Abcam, Cambridge, UK) at 4 °C. On the following day, the cells were washed with 500 μL of PBS and incubated for 1 h in 500 μL of PBS-T containing 3% BSA and a diluted secondary Alexa Fluor 488 AffiniPure Goat Anti-Rabbit IgG (Jackson Immunologicals, West Grove, PA, USA). Mean fluorescence in the captured microscopic images was measured using the ImageJ computer program (NIH, Bethesda, MD, USA).

#### 2.10.2. Hoechst Staining

The cells in 24-well plates were stained with Hoechst staining according to the manufacturer’s protocol (Thermo Fisher Scientific, Waltham, MA, USA). The cells were fixed in 4% paraformaldehyde for 10–15 min. Then, the wells were immersed with Hoechst staining diluted in 1× PBS (1:2000) and incubated in the dark for 30–60 min. The staining intensity in the captured microscopic images was measured using the ImageJ computer program (NIH, Bethesda, MD, USA).

### 2.11. Statistical Analysis

GraphPad prism (GraphPad Software, San Diego, CA, USA) was used to perform all statistical analysis. One-way analysis of variance (ANOVA) was conducted to compare two or more groups, followed by Bonferroni’s correction for multiple comparisons as a post hoc test as appropriate. Results are expressed as mean ± SEM. *p* < 0.05 was considered the criterion for statistical significance. Number of repetitions (*n*) per group are 3, 4 or 5, as described in detail for each experiment.

## 3. Results

### 3.1. LDH Cytotoxicity Assay 

#### 3.1.1. Dose-Response Analysis Using LDH Cytotoxicity Assay

**Figure 1:**
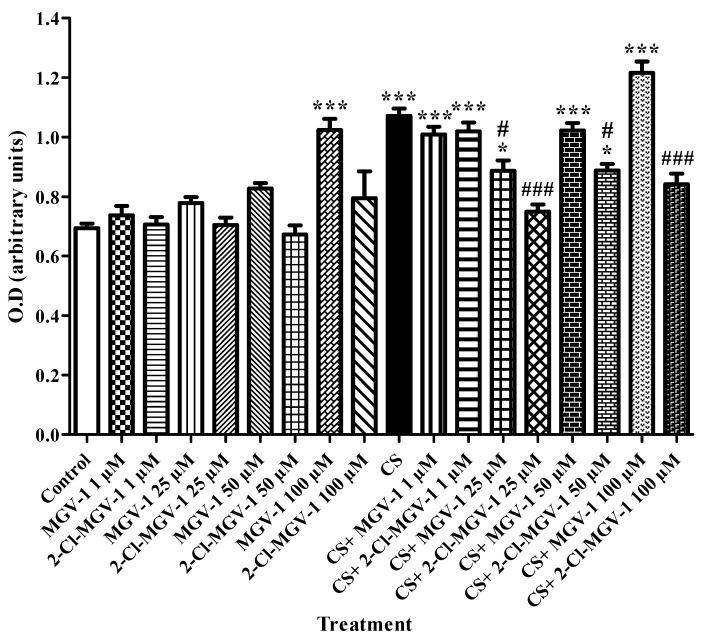
Dose-response analysis of TSPO ligands against CS-induced cytotoxicity in H1299 lung cancer cells. Cytotoxicity was measured by assessment of LDH in the medium using ELISA. Cytotoxicity levels were elevated significantly following CS exposure, while levels of superior protection by 2-Cl-MGV-1 were achieved at a concentration of 25 µM (*n* = 10 in each group). Results are represented as mean ± SEM. * *p* < 0.05 and *** *p* < 0.001 vs. control; # *p* < 0.05 and ### *p* < 0.001 vs. CS. CS = cigarette smoke.

Dose-response analysis was performed by measurement of cytotoxicity levels, as assessed by LDH concentration in the medium. CS exposure of H1299 lung cancer cells induced elevation in cytotoxicity levels by 55% (*p* < 0.001 vs. control; Figure 1). Pretreatment with 2-Cl-MGV-1 at concentrations of 25 and 100 µM significantly prevented 85 and 62% (*p* < 0.001 vs. CS for both; Figure 1) of the CS-induced elevation in cytotoxicity levels, respectively. Pretreatment with MGV-1 (25 µM) and 2-Cl-MGV-1 (50 µM) significantly prevented 49% of the elevation (*p* < 0.05 vs. CS for both; Figure 1) but remained significantly higher than the control values (*p* < 0.05 vs. control; Figure 1). Notably, the application of each of the two ligands separately at increasing concentrations did not cause an alteration in cytotoxicity levels, while cytotoxic effects were observed when MGV-1 was applied at a high concentration of 100 µM (*p* < 0.001 vs. control; Figure 1). Based on these data, a concentration of 25 µM of the ligands was used in this assay.

#### 3.1.2. Cytotoxicity Levels Following Simultaneous- and Post-CS Exposure-Treatment

**Figure 2:**
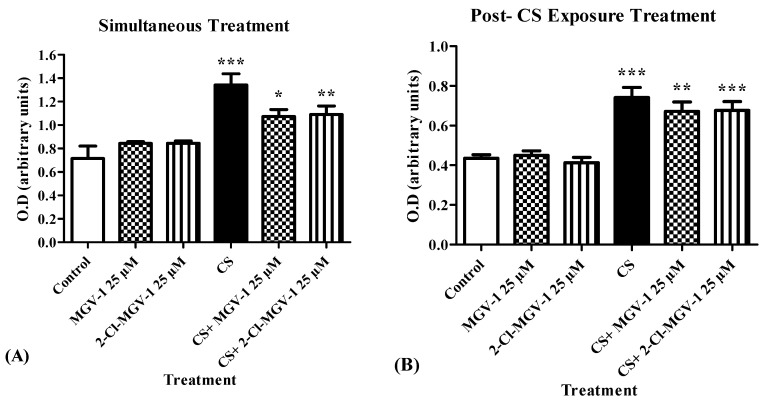
Simultaneous and post-CS exposure treatment with TSPO ligands against CS-induced cytotoxicity in H1299 lung cancer cells. Cytotoxicity levels were elevated significantly following CS exposure, and MGV-1 (25 µM) and 2-Cl-MGV-1 (25 µM) failed to prevent the CS-induced damage when applied as (**A**) simultaneous treatment and (**B**) post-CS exposure treatment. Results are represented as mean ± SEM. * *p* < 0.05, ** *p* < 0.01 and *** *p* < 0.001 vs. control. CS = cigarette smoke.

Cytotoxicity levels were measured following the application of simultaneous and post-CS exposure treatment of MGV-1 and 2-Cl-MGV-1, both at a concentration of 25 µM. In case of simultaneous treatment, CS exposure of 60 min caused a significant increase of 88% (*p* < 0.001 vs. control) in cytotoxicity levels. The application of MGV-1 and 2-Cl-MGV-1 simultaneously with exposure to CS caused a significant prevention of 43% (*p* < 0.05 vs. control) and 40% (*p* < 0.01 vs. control) of the CS-induced cytotoxicity (Figure 2A).

The 60 min exposure to CS, induced a 70% increase (*p* < 0.001 vs. control) in cytotoxicity levels. Post-CS exposure treatment with MGV-1 and 2-Cl-MGV-1, two hours after CS exposure, caused a significant prevention of 23% (*p* < 0.01 vs. control) and 21% (*p* < 0.001 vs. control), respectively, in the CS-induced cytotoxicity (Figure 2B).

#### 3.1.3. Pharmacological TSPO Knockdown-Like Effect Using PK 11,195 as Antagonist

**Figure 3:**
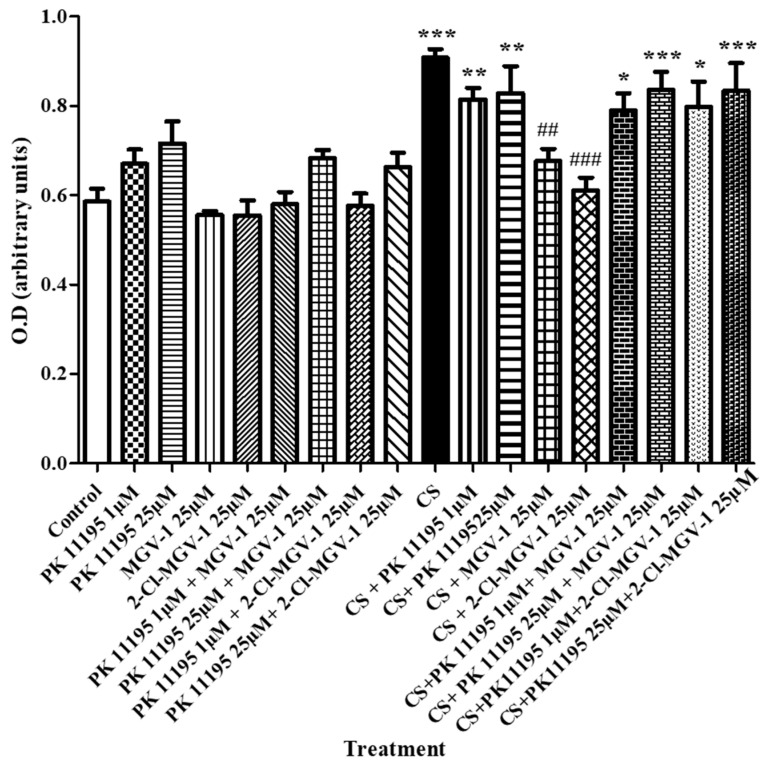
Protective capacity of our TSPO ligands in the pharmacologically induced TSPO knockdown of H1299 lung cancer cells. Cytotoxicity levels were significantly elevated after CS exposure, and MGV-1 (25 µM) and 2-Cl-MGV-1 (25 µM) failed to prevent the CS-induced damage when applied in combination with the antagonistic ligand PK 11,195 at concentrations of 1 and 25 µM. Results are represented as mean ± SEM. * *p* < 0.05, ** *p* < 0.01 and *** *p* < 0.001 vs. control. ## *p* < 0.01 and ### *p* < 0.001 vs. CS. CS = cigarette smoke.

Cytotoxicity levels were measured using the PK 11,195 ligand as an antagonist for mimicking TSPO knockdown-like effect, in combination with our novel ligands MGV-1 and 2-Cl-MGV-1. Sixty minutes of CS exposure resulted in a significant elevation in cytotoxicity levels by 55% (*p* < 0.001 vs. control; Figure 3). The pretreatment with PK 11,195 at concentrations of 1 and 25 µM did not prevent the CS-induced cytotoxic damage (*p* < 0.001 vs. control; Figure 3). The pretreatment with MGV-1 (25 µM) and 2-Cl-MGV-1 (25 µM) significantly prevented the CS-induced cytotoxicity by 73% (*p* < 0.01 vs. CS; Figure 3) and 93% (*p* < 0.001 vs. CS; Figure 3), respectively. The application of the antagonist PK 11,195 at concentrations of 1 and 25 µM in combination with MGV-1 (25 µM) or 2-Cl-MGV-1 (25 µM) as a pretreatment attenuated the protection ability of our ligands against the CS-induced damage. The cytotoxicity levels remained significantly higher by 64% (*p* < 0.05 vs. control; Figure 3) in case of PK 11,195 (1 µM) with MGV-1 (25 µM), by 78% (*p* < 0.001 vs. control; Figure 3) in case of PK 11,195 (25 µM) with MGV-1 (25 µM), by 65% (*p* < 0.05 vs. control; Figure 3) in case of PK 11,195 (1 µM) with 2-Cl-MGV-1 (25 µM) and by 76% (*p* < 0.001 vs. control; Figure 3) in case of PK 11,195 (25 µM) with 2-Cl-MGV-1 (25 µM). All were significant compared to control (Figure 3).

### 3.2. TSPO Levels

#### 3.2.1. Flowcytometry

**Figure 4:**
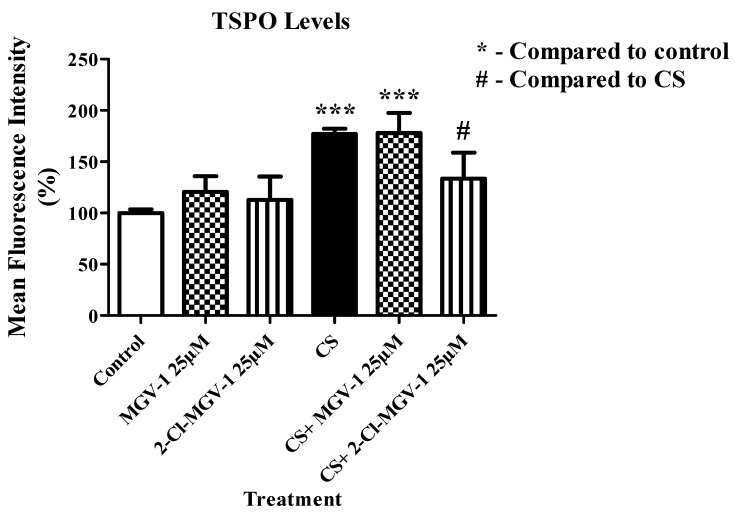
The impact of CS on TSPO levels in H1299 lung cancer cells. The alterations in TSPO levels measured by FACS. Elevation of TSPO expression levels due to CS exposure and the significant protective capacity of the TSPO ligand 2-Cl-MGV-1. Results are represented as mean ± SEM (*n* = 4 in each group). # *p* < 0.05 vs. CS, *** *p* < 0.001 vs. control. CS = cigarette smoke.

TSPO levels were measured using FACS. The application of TSPO ligands MGV-1 and 2-Cl-MGV-1 alone did not affect TSPO levels as compared to the control. CS exposure of H1299 cells led to a significant elevation in TSPO levels by 77% (*p* < 0.001 vs. control; Figure 4). Pretreatment with MGV-1 (25 µM) did not affect significantly the CS-induced TSPO expression (*p* > 0.05 vs. CS; Figure 4). In contrast, pretreatment with 2-Cl-MGV-1 at the same concentration (25 µM) significantly inhibited 57% of the CS-induced elevation in TSPO (*p* < 0.05 vs. CS; Figure 4). The preventive effect was associated with restoration to control levels.

#### 3.2.2. Fluorescence Microscopy

**Figure 5:**
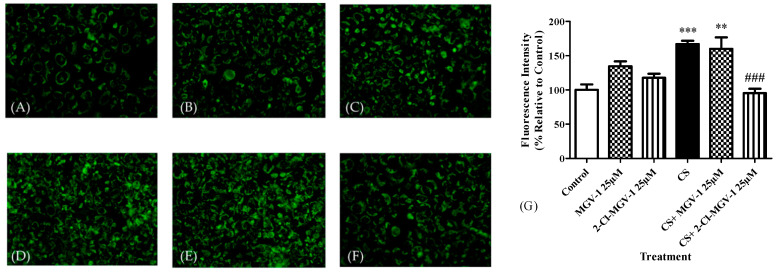
TSPO levels in H1299 lung cancer cells using fluorescence microscopy. The fluorescence intensity using microscopic imaging was measured in (**A**) control group; (**B**) cells pretreated with MGV-1 and exposed to fresh air; (**C**) cells pretreated with 2-Cl-MGV-1 and exposed to fresh air; (**D**) cells exposed to CS without pretreatment; (**E**) cells pretreated with MGV-1 and exposed to CS; (**F**) cells pretreated with 2-Cl-MGV-1 and exposed to CS. (**G**) Representative bar graph. Results are expressed as mean ± SEM (*n* = 4 in each group). ** *p* < 0.01 and *** *p* < 0.001 vs. control, ### *p* < 0.05 vs. CS. CS = cigarette smoke.

Fluorescent labeling of TSPO was performed. The pretreatment with TSPO ligands did not affect TSPO expression (Figure 5B,C), as compared to the control group (Figure 5A). A significant elevation in TSPO expression level by 67% (*p* < 0.001 vs. control) was obtained when cells were exposed for 60 min of CS (Figure 5D). The pretreatment with MGV-1 (25 μM) prevented 10% of the damage induced by CS (*p* > 0.05 vs. CS, Figure 5E), while pretreatment with 2-Cl-MGV-1 (25 μM) totally prevented the cellular damage (*p* < 0.001 vs. CS, Figure 5F).

### 3.3. ADP/ATP Ratio

**Figure 6:**
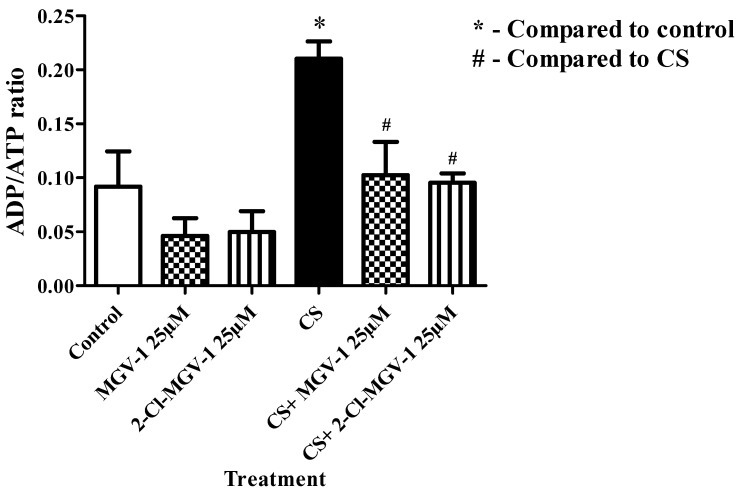
The ability of TSPO ligands against ATP synthase reversal induced by CS in H1299 lung cancer cells. ADP/ATP ratio measured by ELISA. ATP synthase activity was reversed due to CS exposure. The control, MGV-1 and 2-Cl-MGV-1 groups were exposed to fresh air only without exposure to CS. Results are represented as mean ± SEM (*n* = 4 in each group). # *p* < 0.05 vs. CS and *p < 0.05 vs. control. CS = cigarette smoke.

Exposure of H1299 cells to 60 min of CS resulted in a 129% (*p* < 0.05 vs. control) increase in ADP/ATP ratio. Pretreatment of the cells with MGV-1 and 2-Cl-MGV-1 (25 µM) prior to CS exposure significantly prevented 91 and 97% of the increase in ADP/ATP ratio, respectively (*p* < 0.05 vs. CS; Figure 6).

### 3.4. Oxidative Stress Levels

**Figure 7:**
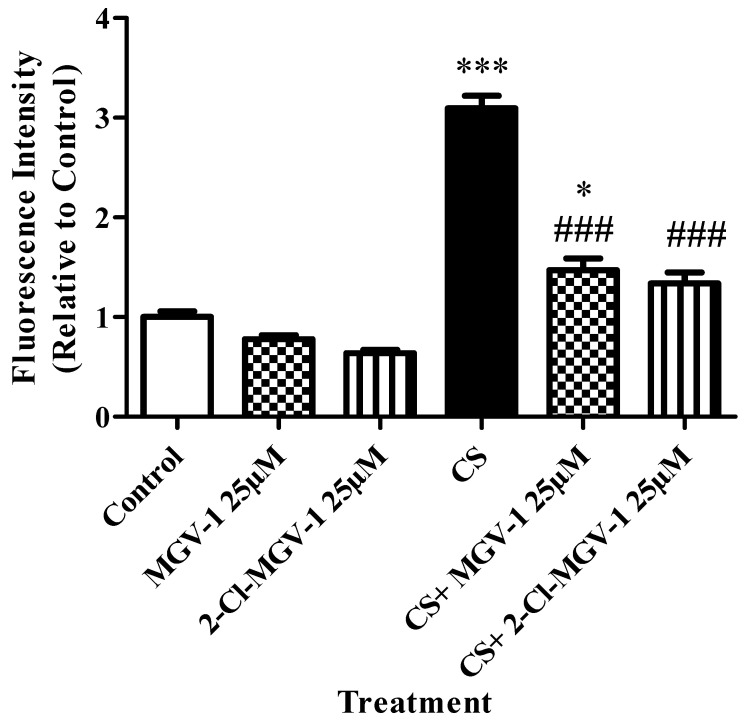
The protective capacity of TSPO ligands against CS-induced ROS production in H1299 lung cancer cells. ROS levels were measured by ELISA. CS significantly elevated oxidative stress levels, while TSPO ligands differentially prevented the CS-induced damage. The control, MGV-1 and 2-Cl-MGV-1 groups were exposed to fresh air only without exposure to CS. Results are represented as mean ± SEM (*n* = 5 in each group). * *p* < 0.05 and *** *p* < 0.001 vs. control, ### *p* < 0.001 vs. CS. CS = cigarette smoke.

CS caused a significant increase in oxidative stress by 209%, as measured with ELISA (*p* < 0.001 vs. control; Figure 7). Pretreatment with MGV-1 (25 µM) significantly attenuated the increase in oxidative stress (77%, *p* < 0.001 vs. CS; Figure 7), but the levels remained significantly higher than the control groups (*p* < 0.05 vs. control; Figure 7). Pretreatment with 2-Cl-MGV-1 (25 µM) significantly inhibited the elevation in oxidative stress (84%, *p* < 0.001 vs. CS; Figure 7) caused by CS, the levels did not differ significantly from the control group. However, as it can be seen, following exposure to CS, the differences between the protective capacity of MGV-1 and 2-Cl-MGV-1 were comparable, 77 and 84%, respectively.

### 3.5. Depolarization of the Mitochondrial Membrane Potential (ΔΨm)

**Figure 8:**
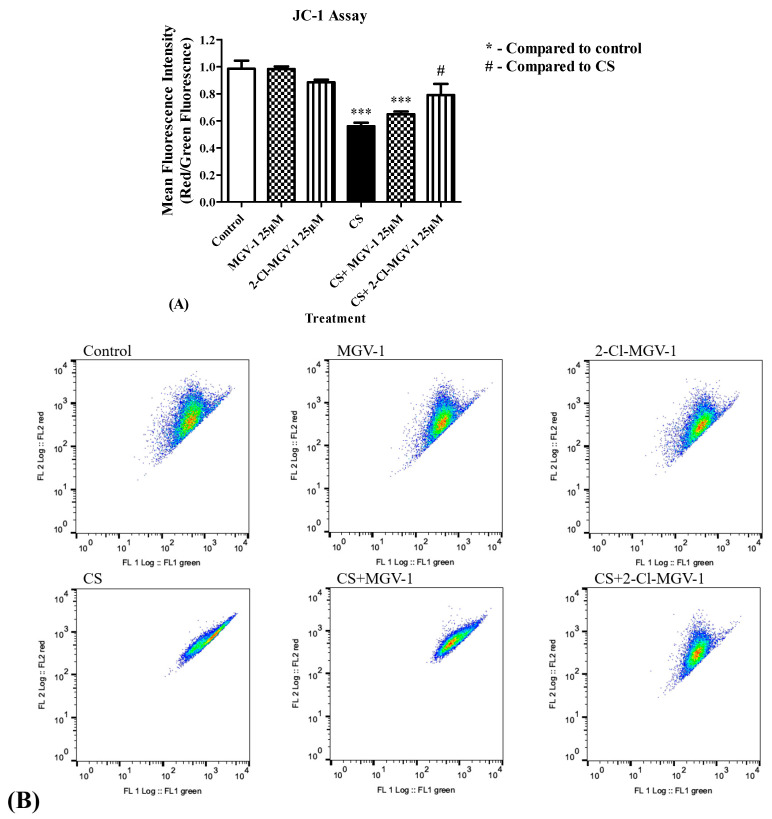
The impact of TSPO ligands on CS-induced mitochondrial membrane potential depolarization in H1299 lung cancer cells. (**A**) FACS was used to quantify the levels mitochondrial membrane potential depolarization following exposure to 60 min of CS. Significant ΔΨm depolarization was seen after exposure to CS and protection by pretreatment with 2-Cl-MGV-1 to control range. (**B**) The results are expressed by the mitochondrial membrane depolarization in the different cellular population in each experimental group. The control, MGV-1 and 2-Cl-MGV-1 groups were exposed to fresh air only. Results are represented as mean ± SEM (*n* = 5 in each group). # *p* < 0.05 vs. CS and *** *p* < 0.001 vs. control. CS = cigarette smoke.

Depolarization of the ΔΨm was measured via calculating the red/green fluorescence intensity ratio as measured by FACS. CS resulted in ΔΨm depolarization by 43% (*p* < 0.001 vs. control; Figure 8A). Pretreatment with TSPO ligands, either MGV-1 or 2-Cl-MGV-1 by themselves, without exposure to CS, did not lead to alterations in the ΔΨm as compared to the control. Pretreatment with the TSPO ligands MGV-1 and 2-Cl-MGV-1 (25 µM) reduced by 21% (*p* > 0.05 vs. CS; Figure 8A) and 53% (*p* < 0.05 vs. CS; Figure 8A) the mitochondrial depolarization caused by CS, respectively. Interestingly, pretreatment with 2-Cl-MGV-1 resulted in restoration of mitochondrial depolarization to the normal range, while MGV-1 did not return to control levels (Figure 8A). The results are demonstrated by the histograms of cellular population in each experimental group (Figure 8B).

### 3.6. Caspase 3, 8 and 9 Levels

**Figure 9:**
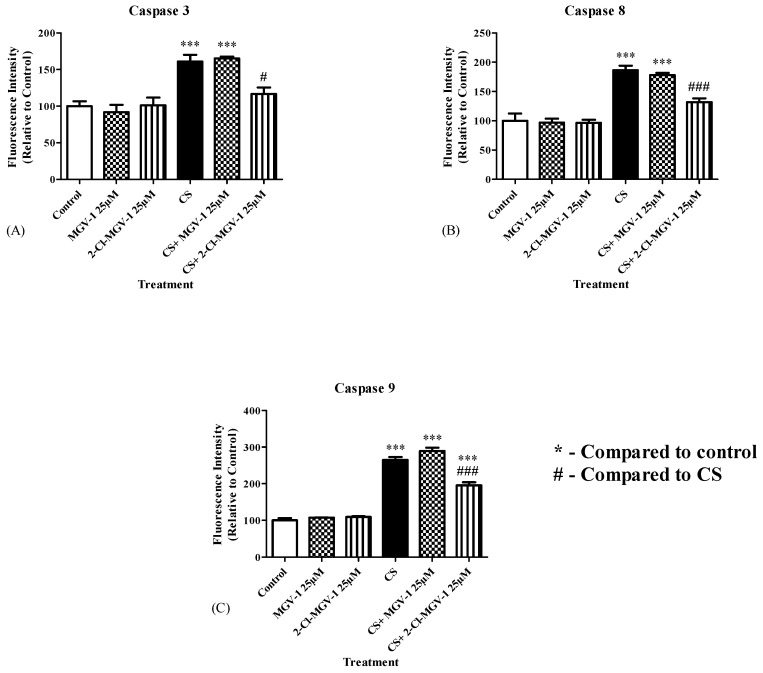
The impact of TSPO ligands on CS-induced increases in caspases 3, 8 and 9 in H1299 lung cancer cells. Caspase multiplex activity assay was used to measure the levels caspase 3 (**A**), 8 (**B**) and 9 (**C**) following CS exposure with or without TSPO ligands. The control, MGV-1 and 2-Cl-MGV-1 groups were exposed to fresh air only. The apoptotic markers, caspases 3, 8 and 9, significantly elevated following CS exposure. Pretreatment with MGV-1 and 2-Cl-MGV-1 attenuated the elevation in the caspases’ levels at various levels. Results are represented as mean ± SEM (*n* = 5 in each group). # *p* < 0.05 and ### *p* < 0.001 vs. CS, *** *p* < 0.001 vs. control. CS = cigarette smoke.

The levels of the apoptotic markers, caspases 3, 8 and 9, were measured using ELISA. In the groups exposed to fresh air after pretreatment with the ligands, caspase 3, 8 and 9 levels remained in the control range. In response to CS exposure, significant increases were detected in caspase 3 (61%, *p* < 0.001 vs. control; Figure 9A), in caspase 8 (86%, *p* < 0.001 vs. control; Figure 9B) and in caspase 9 (165%, *p* < 0.001 vs. control; Figure 9C). Pretreatment with MGV-1 (25 µM) did not present protective effects (*p* > 0.05 vs. CS for all) against the CS-induced elevations in caspase 3 (Figure 9A), caspase 8 (Figure 9B) and caspase 9 (Figure 9C). In contrast, pretreatment with 2-Cl-MGV-1 (25 µM) attenuated by 74% (*p* < 0.05 vs. CS, Figure 7A), 63% (*p* < 0.001 vs. CS, Figure 9B) and 42% (*p* < 0.001 vs. CS, Figure 9C) of the CS-induced increases in caspases 3, 8 and 9, respectively. Furthermore, following pretreatment with 2-Cl-MGV-1, the levels of caspase 3 and caspase 8 were restored to the control range. In contrast, the caspase 9 levels remained significantly higher than the control levels following pretreatment with 2-Cl-MGV-1 (25 µM) (*p* < 0.001 vs. control; Figure 9C).

### 3.7. cAMP Levels

**Figure 10:**
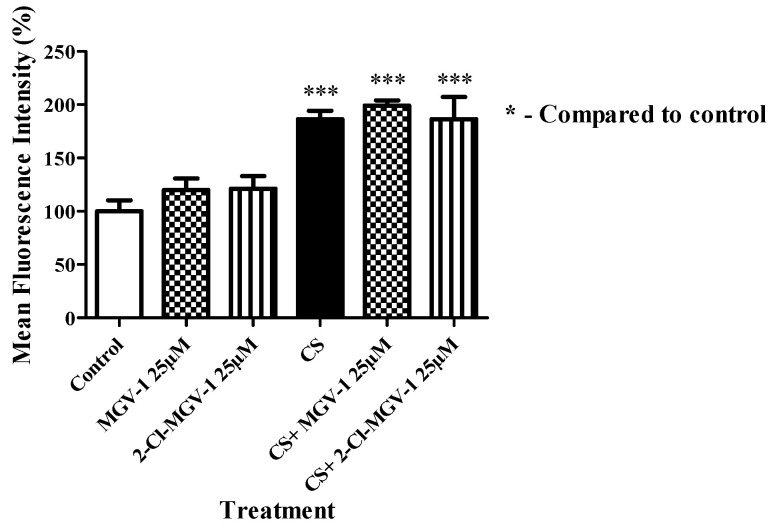
The impact of CS on cAMP levels with and without pretreatment with TSPO ligands in H1299 lung cancer cells. FACS was used to quantify the levels cAMP following exposure to 60 min of CS. The control, MGV-1 and 2-Cl-MGV-1 groups were exposed to fresh air only. Results are represented as mean ± SEM (*n* = 3 in each group). *** *p* < 0.001 vs. control. CS = cigarette smoke.

The levels of cAMP as a pro-apoptotic molecule have significantly increased by 86% (*p* < 0.001 vs. control; Figure 10) following CS exposure. Pretreatment with TSPO ligands before CS exposure did not show any protective effect on the CS-induced elevation in cAMP (*p* > 0.05 vs. CS for all, Figure 10).

### 3.8. Apoptosis Levels

#### 3.8.1. Hoechst Staining

**Figure 11:**
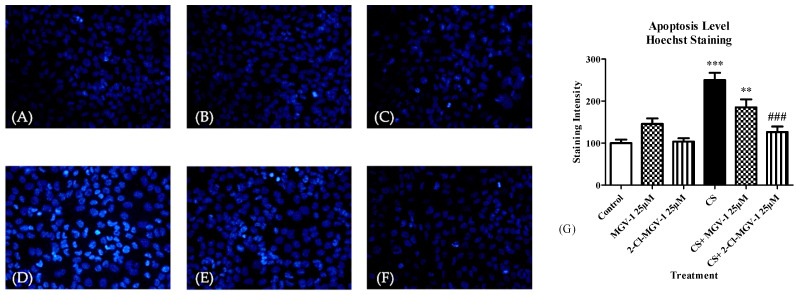
Apoptosis levels in H1299 lung cancer cells as measured by Hoechst staining. The fluorescence intensity of Hoechst staining was determined using microscopic imaging for: (**A**) the control group; (**B**) group of cells pretreated with MGV-1 and exposed to fresh air; (**C**) group of cells pretreated with 2-Cl-MGV-1 and exposed to fresh air; (**D**) group of cells exposed to CS without pretreatment; (**E**) group of cells pretreated with MGV-1 and exposed to CS; (**F**) group of cells pretreated with 2-Cl-MGV-1 and exposed to CS. (**G**) Representative bar graph. Results are expressed as mean ± SEM (*n* = 4 in each group). ** *p* < 0.01 and *** *p* < 0.001 vs. control, ### *p* < 0.001 vs. CS. CS = cigarette smoke.

Hoechst staining was used to stain nuclear DNA. The density of apoptotic cells, reflected by a more condensed nuclear DNA, was significantly increased by 150% after exposure of 60 min to CS (*p* < 0.001, Figure 11D) as compared to the control group (Figure 11A). The pretreatment with the TSPO ligand MGV-1 (25 μM) prevented 43% of the apoptotic cell death induced by CS (*p* > 0.05 vs. CS, Figure 11E). Pretreatment with 2-Cl-MGV-1 at similar concentration (25 μM) significantly prevented 83% of the damage (*p* < 0.001 vs. CS, Figure 11F).

#### 3.8.2. Fluorescence Activated Cell Sorting (FACS) Assay

**Figure 12:**
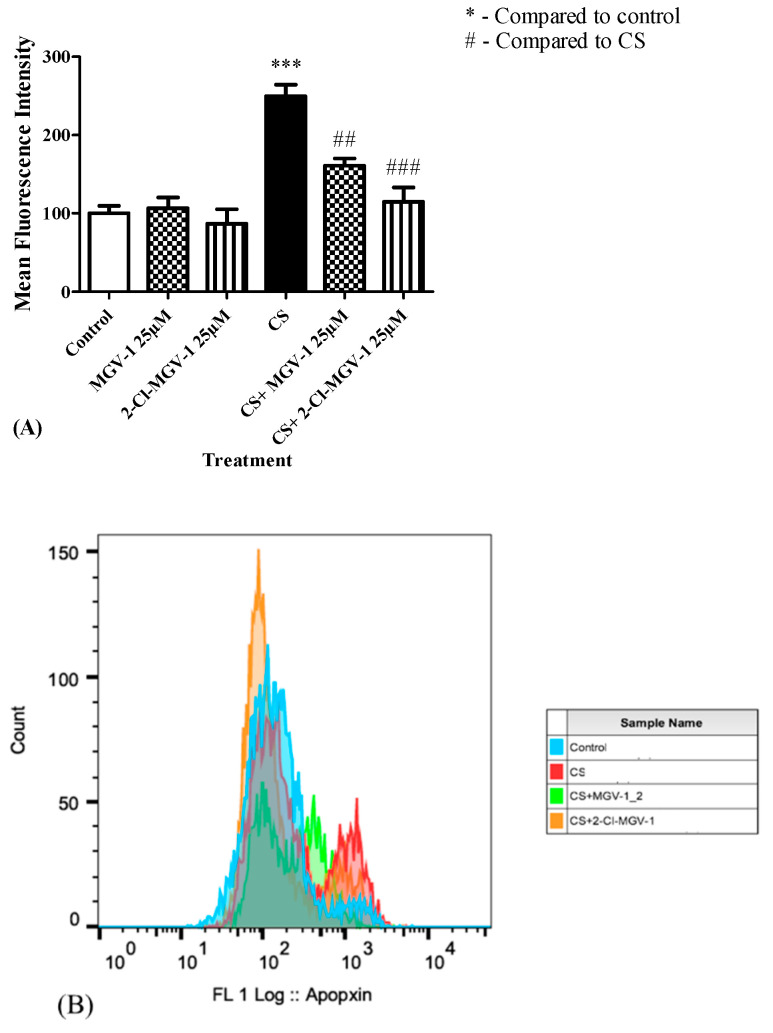
Apoptosis levels in H1299 lung cancer cells as measured by FACS. The fluorescence intensity was determined using FACS in (**A**) a bar graph and (**B**) a representative histogram. Results are expressed as mean ± SEM (*n* = 5 in each group). *** *p* < 0.001 vs. control, ## *p* < 0.01 and ### *p* < 0.001 vs. CS. CS = cigarette smoke.

Apoptosis level was measured using FACS. CS exposure for 60 min resulted in an increase in the level of apoptosis by 150% (*p* < 0.001 vs. control; Figure 12A). The pretreatment application of our TSPO ligands, MGV-1 and 2-Cl-MGV-1 (both at 25 µM), prevented the CS-induced apoptotic cell death by 60% (*p* < 0.01 vs. CS; Figure 12A) and by 91% (*p* < 0.001 vs. CS; Figure 12A), respectively. The pretreatment with MGV-1 and 2-Cl-MGV-1 did not cause cytotoxic effects when applied alone, with no exposure to CS (Figure 12A). The increase in apoptotic cell death levels and the protection ability of our TSPO ligands is also reflected in the representative histogram below (Figure 12B).

### 3.9. Association between TSPO Expression and Apoptosis

**Figure 13:**
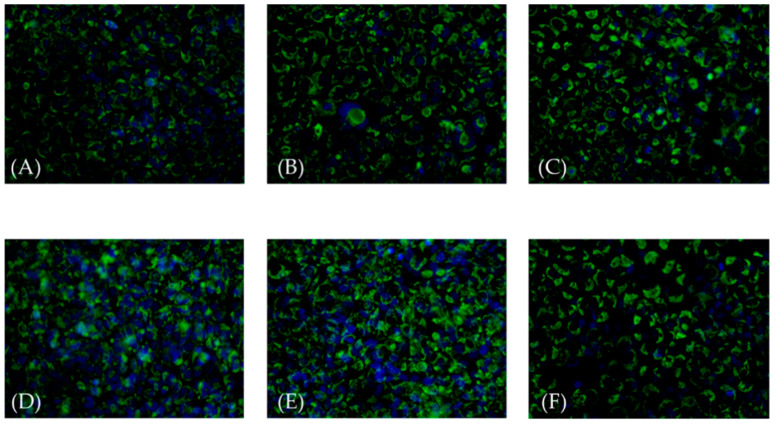
Merged microscopic images of TSPO expression and apoptosis in H1299 lung cancer cells. The microscopic images representing the fluorescence intensity of both Hoechst staining (blue) and of TSPO expression (green) were merged, demonstrating the association between TSPO and apoptotic cell death: (**A**) the control group; (**B**) group of cells pretreated with MGV-1 and exposed to fresh air; (**C**) group of cells pretreated with 2-Cl-MGV-1 and exposed to fresh air; (**D**) group of cells exposed to CS without pretreatment; (**E**) group of cells pretreated with MGV-1 and exposed to CS; (**F**) group of cells pretreated with 2-Cl-MGV-1 and exposed to CS. CS = cigarette smoke.

The association between TSPO expression and apoptotic cell death is represented by the merged TSPO expression with apoptosis microscopic images. Pretreatment with TSPO ligands alone did not alter the TSPO expression levels. Increased TSPO following exposure to CS is accompanied by a corresponding elevation in apoptotic cell death (Figure 13D). Similarly, reduction in apoptotic cell death in the groups pretreated with the TSPO ligands prior to CS exposure is accompanied by reduction in TSPO expression levels (Figure 13E,F).

## 4. Discussion

**Scheme 2:**
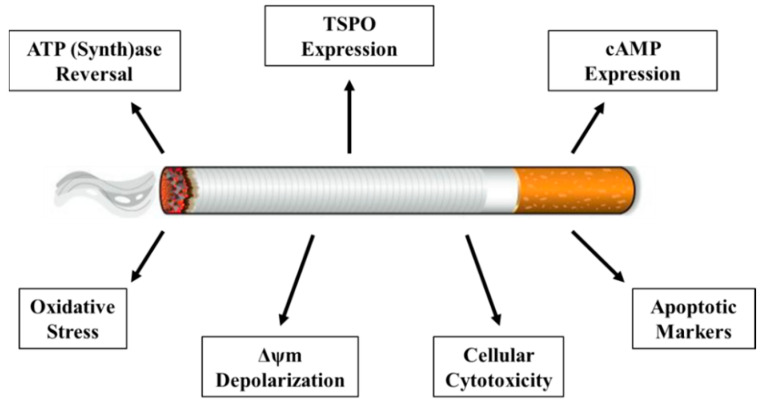
Cigarette smoke (CS) impact on TSPO and TSPO-related cellular damage.

In this study, we investigated the protective capacity of the two novel TSPO ligands, MGV-1 and 2-Cl-MGV-1, in the prevention of the cytotoxic effects of cigarette smoke (CS) on TSPO-related mitochondrial processes (Scheme 2). Previously, it was shown that TSPO expression levels were significantly elevated only after 60 min of CS exposure. In addition, mitochondrial processes including ATP synthase activity, ROS generation, mitochondrial membrane potential and cell death, mainly apoptotic cell death, was significantly altered following prolonged exposure to CS [22]. The association between TSPO and cancer was previously demonstrated and showed elevated TSPO expression levels in case of myeloid and tumor cells, as compared to moderate expression levels in healthy brains [15,16]. In the current study, the application of our TSPO ligands did not affect TSPO expression levels when applied alone, indicating a lack of toxic impact on the H1299 cells. In contrast, TSPO expression levels were significantly increased by 77% (*p* < 0.001 vs. control) following 60 min of CS exposure, and the novel ligand 2-Cl-MGV-1 was effective in the prevention of this elevation (Figure 4). This CS-induced increase in TSPO expression correlates with the upregulation of mitochondrial processes involved in cellular damage and, thus, may reflect cellular damage (Figure 4 and Figure 5). Additionally, reversal of ATP synthase activity occurs, leading to increased cellular ADP/ATP ratio, which indicates a pathological state of the cells. These processes, along with other TSPO-related processes, were altered at various degrees following CS exposure, and these alterations were attenuated by pretreatment with the TSPO ligands. On the other hand, the application of our TSPO ligands as co-treatment (simultaneous treatment) or post-treatment did not achieve a significant protective capacity against the CS-induced cellular damage (Figure 2). Furthermore, PK 11195-related pharmacological TSPO knockdown-mimicking effect led to diminished protection of our TSPO ligands against the CS-induced cytotoxicity (Figure 3) [42], indicating the role of TSPO in the protective effect of the ligands. The underlying mechanism behind the elevation in TSPO expression in tumors and in the neighboring inflammatory regions remains unclear [15]. The protective impact of our TSPO ligands and the results reported in this study further indicate the role of TSPO in cell damage and disease development. The attenuation of the CS-induced damages by the TSPO ligands may implicate a potential role in the prevention of pulmonary diseases. The application of 2-Cl-MGV-1 alone with exposure to fresh air only led to adverse impact on some cellular markers, such as TSPO expression, ΔΨm depolarization and cytotoxicity levels; however, these effects were statistically non-significant. In contrast, 2-Cl-MGV-1 achieved a significant protective effect on the same biological processes (Figure 1, Figure 4, Figure 5 and Figure 8).

One of the main intracellular functions of TSPO is its regulatory role in apoptotic cell death [12,13]. Caspase-dependent apoptosis was previously demonstrated to be sensitive to TSPO ligands [24,25,43,44]. In this study, caspase-dependent apoptosis was expressed by the significant elevation in cell death levels along with the apoptotic markers caspases 3, 8 and 9 following 60 min of CS exposure. The elevation of these apoptotic markers was prevented when the ligands were administered as pretreatment 24 h prior to CS exposure (Figure 9). Quinazoline derivatives previously expressed antimicrobial [45,46], cholinesterase inhibition [47], anti-inflammatory [48] and anticancer [49] effects. In the perspective of our study, it appears that our two quinazoline scaffold-based TSPO ligands are able to prevent cell death as shown also by the prevention of LDH elevation (Figure 1). Additionally, this indicates the regulatory role of our TSPO ligands in apoptotic cell death process (Figure 9, Figure 11 and Figure 12). It is of note that the same TSPO ligands did not prevent all pro-apoptotic processes, and elevation in pro-apoptotic cAMP was not inhibited by the ligands (Figure 10). In the present study, two methods for measuring apoptosis were used. The fluorescence intensity in the microscopic images reflects the density of nuclear DNA, thus, expressing to some extent apoptotic cell death levels. On the other hand, FACS using apopxin staining to measure apoptotic cell death levels in the different experimental groups, in order to further validate the findings shown by the first experiment. However, in the microscopic images, cell count may be limited, since some cells are not stained due to their non-apoptotic state.

A previous study has shown elevated necrotic cell death following prolonged exposure time to CS [22]. The TSPO ligands partially inhibited the cytotoxic effect of CS, as assessed by LDH levels. The inhibition of the CS-induced TSPO elevation (Figure 4 and Figure 5), increases in several caspases (Figure 9), depolarization of ΔΨm (Figure 8), LDH enzyme elevation (Figure 1) and the elevation in apoptotic cell death levels (Figure 11 and Figure 12) indicates the possible anti-apoptotic activity of TSPO and its lack of effect on necrotic cell death, since a major protective effect was seen by the impact of our ligands on several apoptotic markers. Our findings are supported by a previous study that demonstrated the impact of smoking on apoptotic cell death and associated mitochondrial processes, which are consistent with the results of our current study. However, in the Cui et al. study, the exposure to CS is achieved by the cells to CS dissolved in medium, while in our current study, we exposed the cells directly to CS, similar to actively smoking lungs [50]. The association between TSPO and apoptotic cell death in this study is demonstrated by the correlation between alterations in TSPO expression levels and the apoptosis-related cellular processes such as depolarization of ΔΨm, upregulation of caspases 3, 8 and 9 and Hoechst staining (Figure 8, Figure 9, Figure 11 and Figure 12). Nevertheless, the use of alternative assays for assessing apoptosis and TSPO expression (e.g., qRT-PCR and Western blot) could support and validate our results. Moreover, the relationship between upregulation of TSPO and lung cancer is unclear [51].

Furthermore, the superior anti-inflammatory role of the low-affinity TSPO ligands MGV-1 and 2-Cl-MGV-1 over the high-affinity PK 11,195 ligand was previously demonstrated in our lab [52]. These anti-inflammatory effects of TSPO ligands [15] may be relevant to the progression of tumor cells and the inflammation surrounding tumors. Therefore, treatment with the low-affinity ligands may be safer than the treatment with the high-affinity ligand PK 11,195 [52].

There is a growing controversy concerning the TSPO-related functions; thus, a dose-response effect study can clarify the specificity of ligands effect. Unfortunately, the ligands in the present study were used at a single concentration (25 µM) that is more than 30 folds higher than calculated Ki. This concentration was chosen based on a previous dose-response study [38]. However, that study [38] was performed using two other cell lines (U118MG and PC12) and only two parameters that were previously measured (ΔΨm and LDH activity) were also measured in the current study. Hence, in the absence of a dose-response analysis, it is possible that the mild significant effects reported in the present study do not reflect the maximal expected ligand effect at lower or higher concentrations.

The exact mechanism via which CS induces its cytotoxic effects and the protective mechanism achieved by MGV-1 and 2-Cl-MGV-1 remain unclear. Further whole gene expression studies and analysis of cellular-molecular pathways are needed to identify the intracellular pathways that are responsible for the inhibitory effect of our TSPO ligands on apoptotic cell death in lung cells.

The difference between the two low-affinity ligands was seen along the different experiments. In particular, the superior protective capacity of 2-Cl-MGV-1 over MGV-1 was seen in prevention of the CS-induced elevation in TSPO expression elevation, depolarization of ΔΨm and upregulation of caspases 3, 8 and 9. On the other hand, MGV-1 was effective in prevention of the CS-induced cellular cytotoxicity, oxidative stress and ATP synthase reversal. However, this advantage may be related to differences in effective dose, which was not explored in this study. In the present study, we used PK 11,195 as an antagonist of TSPO; however, some studies used PK 11,195 as a TSPO agonist, and it was previously described that TSPO ligands, including PK11195, could have agonistic or antagonistic effects depending on the studied function [42,53]. Moreover, PK 11,195 was also reported to induce TSPO-independent effects, especially concerning ATP-synthase [54]. However, in our studies, we have shown that our ligands MGV-1 and 2-Cl-MGV-1 are competitors of the PK 11,195 binding to TSPO [55]. Furthermore, previous studies have demonstrated antagonistic activity of the PK 11,195 at the TSPO [42]. In the future studies, we aim to perform siRNA extinction of TSPO (even partially) or other knock-down strategies (shRNA, Crispr-cas9), as well as to extend the impact of PK 11,195 on all the functions that were tested in the present study. Furthermore, in the current study, we assessed the impact of the TSPO ligands on one cell line. We intend in the future to extend our experiments to other cell lines, as well as assessment of the impact of a high-affinity TSPO agonist on cellular functions.

## 5. Conclusions

In conclusion, the current data demonstrate the role of the low-affinity TSPO ligands MGV-1 and 2-Cl-MGV-1 in protection against cell death and mitochondrial damage of H1299 lung cancer cells exposed directly to CS. The current findings may have clinical implications for the possible use of MGV-1 and 2-Cl-MGV-1 in the prevention (on daily or periodic administration in smokers) and possibly in the treatment (applied if disease already occurs in smokers) of CS-associated pulmonary damage and diseases, such as lung cancer and COPD. Further cellular and molecular experiments are needed to assess the therapeutic ability of our TSPO ligands, besides their protective role when applied as a pretreatment. In addition, further studies with knockout and knockdown cells are required to confirm or disprove the role of TSPO in the attenuation of apoptotic cell death, and the prevention of CS-induced cellular damages achieved by the novel TSPO ligands. The current study demonstrated a beneficial effect of a single in vitro pretreatment with our TSPO ligands on lung cell damage induced by CS; however, the implications for long-term preventive treatment are as yet unclear.

## Data Availability

Not appliacable.
